# Recovery of Valuable Aromas from Sardine Cooking Wastewaters by Pervaporation with Fractionated Condensation: Matrix Effect and Model Validation

**DOI:** 10.3390/membranes12100988

**Published:** 2022-10-11

**Authors:** M. João Pereira, Manuela Pintado, Carla Brazinha, João Crespo

**Affiliations:** 1 LAQV-REQUIMTE, Department of Chemistry, NOVA School of Science and Technology, FCT NOVA, Universidade NOVA de Lisboa, 2829-516 Caparica, Portugal; 2 CBQF/Escola Superior de Biotecnologia, Universidade Católica Portuguesa, Rua Diogo Botelho, 1327, 4169-005 Porto, Portugal

**Keywords:** modelling of organophilic pervaporation, vacuum fractionated condensation, aroma recovery, valorisation of canning industry effluents, removal of off-flavours

## Abstract

Due to the lack of studies addressing the influence of real food matrices on integrated organophilic pervaporation/fractionated condensation processes, the present work analyses the impact of the real matrix of sardine cooking wastewaters on the fractionation of aromas. In a previous study, a thermodynamic/material balance model was developed to describe the integrated pervaporation—a fractionated condensation process of aroma recovery from model solutions that emulate seafood industry aqueous effluents, aiming to define the best conditions for off-flavour removal. This work assesses whether the previously developed mathematical model, validated only with model solutions, is also applicable in predicting the fractionation of aromas of different chemical families from real effluents (sardine cooking wastewaters), aiming for off-flavour removals. It was found that the food matrix does not influence substantial detrimental consequences on the model simulations, which validates and extends the applicability of the model.

## 1. Introduction

The large majority of studies performed for aroma recovery by pervaporation have been accomplished using model solutions [1]. The use of model systems is effective for a simple and detailed analysis of process performance and optimisation. However, model solutions cannot reproduce all the complex varieties of constituents of the feed stream, with diverse concentrations and chemical and organoleptic properties which contribute to the overall aroma profile [2]. The pervaporation of real feed mixtures should also be studied because the concentration of volatiles is usually lower than in model solutions, due to potential interferences of lipids and proteins in the aroma profile [3], which is mostly neglected when studying model solutions. However, there are still a few studies that use real feeds [3,4,5,6].

In a previous study, Pereira et al. [7] proposed a mathematical model for the pervaporation-fractionated condensation aiming at the recovery of aromas free from off-flavours using a model solution that mimicked seafood cooking wastewaters. This model allows for simulating the mass and composition of each compound in the condensers arranged in a series, mousing as input information the permeate fluxes of each aroma under study (obtained experimentally), operating conditions used in the process, and thermodynamic parameters of each aroma. The model is based on the mass balances and thermodynamic equilibrium in each condenser.

For many years, the production of commercial seafood flavourings used solid by-products. Nowadays, seafood cooking water has emerged as a promising source for producing “natural-like” aroma concentrates, valuable for the food and feed market sectors [8]. The presence of off-flavours in the agro-industrial effluents composition is one of the constraints associated with the valorisation of aromas. Even though they are frequently innocuous, off-flavours might degrade the quality of a food product, which can be quite expensive for the food and beverage sectors. The Maillard reaction or lipid oxidation, which produces numerous food smells and certain off-flavours, develops during thermal processing. The need for non-thermal processes or the use of gentler conditions is growing as a result [9]. In this work, the fractionation and separation of desirable target aromas from off-flavours are explored, benefiting from both the membrane’s intrinsic selectivity and the selectivity of fractionated condensation consecutive steps.

The main objective of this work is to study the effect of the matrix on the aroma recovery from sardine cooking wastewaters by the integrated process of organophilic pervaporation/fractionated condensation, assuring off-flavour removal. Concretely, the objective is to validate the mathematical model previously developed for model solutions, extending it to apply to a real matrix, a complex sardine cooking wastewater. If the model correctly predicts the fractionation of the different aromas, despite the complexity of the real solution, the applicability of the model will increase significantly, opening opportunities for use with other real matrices.

## 2. Materials and Methods

The sardine cooking wastewater was kindly provided by the company A Poveira S.A. (Laúndos, Portugal). This effluent is the result of steaming the fish for 7 min at 100 °C. An acorn extract with antioxidant properties was added to the sardine cooking wastewater at the outlet of cooking chambers at a 1% (*v*/*v*) concentration to prevent lipid oxidation and suppress aroma deterioration. The effluent was collected, transported, and stored at −20 °C until needed.

The experimental setup and analytical methods of study were the same as described in previous studies [7,10].

A radial flow flat module (GKSS, Germany) was employed, presented, and discussed in detail in Schafer [11]. The membrane used was a PervapTM 4060 (DeltaMem AG, Switzerland), an organophilic dense membrane with a membrane area of 10^−2^ m^2^. The active layer of polydimethylsiloxane (PDMS) was shown to have an excellent performance for the permeation of organic compounds by pervaporation, as well as a good affinity for seafood aromas [12,13]. 

The operation conditions applied in this study were the optimised conditions obtained previously in the studies performed with a model solution [7]. According to these, the permeate pressure applied was 1500 Pa. The temperature of the first condenser T1, condens was set at −100 °C, and the temperature of the second condenser *T_*2*,condens_* was at −196 °C. 

At the end of the trials, the membrane used was rinsed with a known amount of water at room temperature, and the content of lipids, proteins, and aromas present in this solution was characterised according to the methods described in Pereira et al. [10].

## 3. Results

### 3.1. Characterisation of Sardine Cooking Wastewaters

Alcohols, aldehydes, and ketones are part of the aroma profile of the sardine cooking wastewaters, as revealed by solid-phase microextraction followed by gas chromatography mass spectrometry (SPME/GC-MS). The overall aroma profile of sardine cooking wastewaters is presented in Table 1, and it is identical to the aroma profile of sardines investigated by other researchers [14,15]. Some chemical markers were selected to study the effect of the matrix in this process, which are 1-penten-3-ol and 1-octen-3-ol, as alcohols; heptanal, (*E,E*)-2,4-heptadienal, (*E,Z*)-2,6-nonadienal, as aldehydes; and 2-nonanone as ketone. These chemical markers were selected based on the main groups of chemicals present in sardine cooking wastewaters, with diverse organoleptic properties. The main compound present in higher concentrations was 1-penten-3-ol.

### 3.2. Pervaporation-Fractionated Condensation Processing of Sardine Cooking Wastewaters

The permeate was generated through pervaporation experiments with sardine cooking wastewaters under upstream operating conditions described in the previous section. The total permeate fluxes obtained in the seafood model solution experiments using the same operating conditions were 889.84 g/m^2^.h and 731 g/m^2^.h with sardine cooking wastewaters. This lower value for the permeate flux was expected due to the total lipid (28.13 ± 2.84 g/100 g) and protein content (25.38 ± 1.95 mg/mL) of the sardine wastewater sample [10]. The presence of lipids and proteins in the feed medium might lead to interactions with aroma compounds present and also to some degree of fouling of the pervaporation membrane.

Table 2 shows the individual fluxes [mol/(m^2^.s)] and the permeabilities [mol/(m.s.Pa)] to the aromas under study, as well as the separation factors obtained (calculated against water).

The important and main alcohol 1-penten-3-ol, responsible for the aroma of fresh marine products, is generated from polyunsaturated fatty acids [15]. 1-Penten-3-ol presents the highest values for the individual flux (*Ji*) and permeability (*Li*). However, the off-flavour (*E2*, *Z6*)-nonadienal shows a close permeate flux and the highest separation factor, which reinforces the importance of conjugating fractionated condensation to the pervaporation process to enable the off-flavour’s removal.

At the end of the process, to better understand the effect of the matrix in the pervaporation process, the content of the total proteins and lipids that remained adsorbed to the membrane, as well as the aroma content in this adsorbed layer, were analysed by Lowry and Bligh and Dyer’s methods, respectively. There was no gel formation on the membrane surface, and indeed, the protein content in the membrane was quite residual (6.15–8.46 µg/m^2^), only slightly more relevant in terms of lipids showing 1.3–2 μg/m^2^. Concerning the aromas, a small number of aromas remained in the membrane in a very small concentration: only 2-nonanone and 1-octen-3-ol were found in residual concentrations of 10 and 20 µg/m^2^, respectively.

#### Model Validation for the Real Sardine Cooking Wastewater

The thermodynamic/material balance model was developed to simulate the recovery of aromas at a given permeate pressure employing fractionated condensation with two condensers in a series, supported by an efficient and optimised fractionated condensation (see the complete explanation by Pereira et al. [7]). In short, starting from simple experimental inputs such as the (i) permeate flux of each aroma present in the system, (ii) thermodynamic parameters (for each compound in the feed: Antoine constant and activity coefficient at infinite dilution), and (iii) operation conditions of downstream pressure and temperature, it is possible to simulate the composition of the condensates obtained in the sequential condensers. Through a system of equations that describe the thermodynamic equilibrium conditions and with the support of required material balances, we can select the best operating conditions to achieve the best separation of desirable flavours from off-flavours. In the end, the expressions for calculating the percentage of condensation of water and aroma(s) in the first condenser are obtained, respectively, by Equations (1) and (2).

(1) $$\%condens_{w1}=1-\frac{n_{inert}}{n_{w0}}\cdot \frac{p_{vw\left(T_{1,\;condens}\right)}}{p_{perm}-p_{vw\left(T_{1,\;condens}\right)}}$$

(2) $$\%condens_{aroma1}≅1-\frac{n_{inert}}{n_{aroma0}}\cdot \frac{\varkappa_{aroma1}\cdot \gamma_{aroma1}^{\infty }\cdot p_{varoma\left(T_{1,\;condens}\right)}}{p_{perm}-p_{vw\left(T_{1,\;condens}\right)}}$$

where *n_inert_* is the inert gas molar flow rate in the stream, *P_v_* is the saturation vapour pressure of water or aroma, *pperm* is the permeate pressure applied to the system, *n_w_ _or aroma0_* is the molar flow rate before the first condenser, ϰ*_w or aroma_* is the molar fraction in the feed, and ϒ^∞^_aroma_ is the infinite activity coefficient of the aroma.

**Figure 1:**
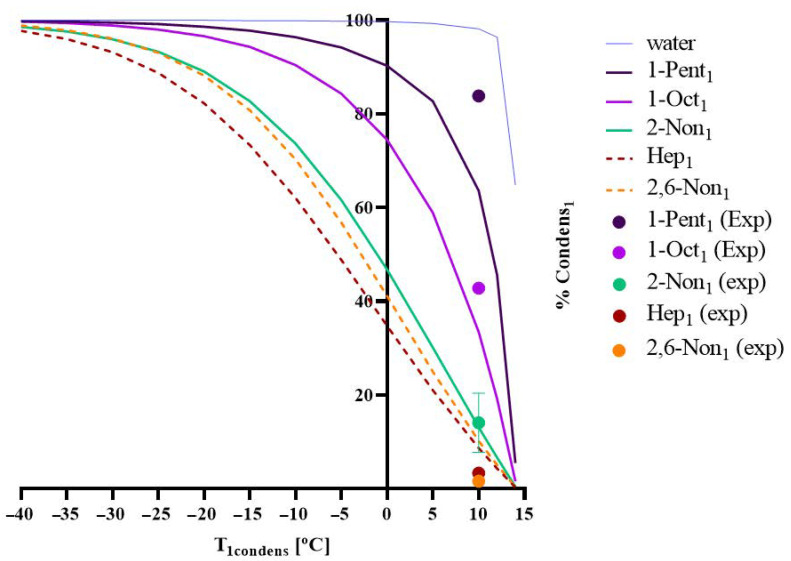
A model simulation was obtained for the sardine cooking wastewaters with experimental validation for five different aroma presents. Percentage of condensation of each compound (water, 1-penten-3-ol, 1-octen-3-ol, heptanal, 2,6-nonadienal, and 2-nonanone) in the 1st condenser (*%Condens_i*1*_*) as a function of the temperature of the same condenser (*T_*1*,condens_*). Operating conditions: Pervap 4060 membrane; *T_feed_* = 60 °C; *p_perm_* = 1500 Pa; lines refer to simulated values and dots to experimental data, which were analysed in triplicate.

The model developed was applied for a permeate pressure of 1500 Pa, where the percentage of compound *i* that is condensed/recovered in the first condenser, *%Condens_i*1*_*, was predicted for different values of *T_*1*,condens_* [°C]. Figure 1 shows the simulations obtained for each aroma present in the sardine cooking wastewater and the experimental values acquired, in terms of *%Condens_i*1*_* (the fraction of each chemical compound *i* that condenses in the first condenser) versus the temperature of the condenser, *T_*1*,condens_*.

Figure 1 reveals a good adherence between the experimental and the simulated results of *%Condens_i*1*_* as a function of the temperature of the condenser and, consequently, for the composition of condensates. This result means that, although the real medium composition is much more complex than the model solution previously studied, it is not necessary to modify the thermodynamic/material balance model used, which can be applied with success to evaluate if a given fractionation of aromas (such as the fractionation between target aromas and off-flavours) can be achieved.

Under the experimental conditions used in this work, 1500 Pa of permeate pressure and *T_*1*,condens_* [°C] of 10 °C (see Figure 1), a good off-flavour removal was achieved, with the partial retention of off-flavours in the 1st condenser lower than 3% for heptanal and 1.6% for (*E2*, *Z6*)-nonadienal. These retention values correspond to an off-flavour concentration of 0.02 and 0.67 mg _off-flavours_/Kg_condensate_ of heptanal and (*E2*, *Z6*)-nonadienal, respectively, in the 1st condensate. In a conclusion, in terms of aroma quality, the condensate recovered in the first condenser is reduced in off-flavours. Both off-flavour concentrations are below their threshold (limit of human olfactive perception) of 0.60 and 0.70 mg/L of heptanal and (*E2*, *Z6*)-nonadienal, respectively. On the other hand, it should be recognised that the recovery of desirable aromas in the first condenser is not complete, not assuring off-flavour removal: 84% of 1-penten-3-ol is recovered in the first condenser, but 43% of 1-octen-3-ol is recovered, and only 14% of the ketone 2-nonanone is recovered.

This model proves to be an excellent tool to simulate the percentage of the condensation of each aroma in each condenser at a particular downstream pressure and condenser temperature, as well as the resulting condensate composition. It enables the comparison and definition of fractionated condensation procedures based on the goal of a given industrial process.

## 4. Conclusions

The integrated process of pervaporation-fractionated condensation has proven to be a potential approach for the valorisation of canning industry effluents by the recovery of valuable aromas.

The model used shows a good match between experimental and predicted values, despite the high heterogeneity of the sardine cooking wastewater. The effect of the matrix did not demonstrate significant negative effects on the model simulations, making possible its use without further modification.

The model applied and validated for sardine cooking wastewaters proved to be a useful tool to predict the fractionation of aroma compounds, here illustrated by the removal of off-flavours to obtain aroma products with potential commercial value. Additionally, due to the range of chemical families evaluated, this model represents a tool that might be easily applied to other real matrices, aiming at the recovery of surplus aromas from a circular economy perspective.

## Figures and Tables

**Table 1:** Aroma compounds identified in Sardine cooking wastewaters.

Aroma Compounds	Area *	[Ci] (ppm) *	Aroma Compounds	Area *	[Ci] (ppm) *
**Aldehydes**			**Alcohols**		
Hexanal	123246866		1-Penten-3-ol	79964041	0.100
Heptanal	28381616	0.006	1-Octen-3-ol	181778114	0.008
2-Hexenal, (E)-	58129859		(5Z)-Octa-1,5-dien-3-ol	86516933	
Octanal	16922895		2-Ethylhexanol	7501041	
Nonanal	79342642		1-Octanol	590437999	0.100
2-Octenal, (E)-	39611501		1-Penten-3-ol	79964041	0.008
2,4-Heptadienal, (*E,E*)-	88078708		1-Octen-3-ol	181778114	
2-Nonenal, (E)-	21644129	0.011	**Ketones**		
2,6-Nonadienal, (*E,Z*)-	92347778	0.044	2-Nonanone	44929994	0.001
2-Decenal, (E)-	12169097		3,5-Octadien-2-one	113901449	
**Sulphur compounds**			2-Undecanone	8019036	
Trans-2-(2-Pentenyl)furan	46428766		**Acids**		
			Hexanoic acid	89583410	

* Mean values of integration peak areas for all compounds identified and the concentration (ppm) of chemical markers. The aromas were identified by comparing their retention indices relative to C8–C20 n-alkanes and their mass spectra to those in the NIST Library Database. Quantification was performed with calibration curves of the pure standards, evaluated under the same circumstances. Underlined compounds are off-flavours.

**Table 2:** Experimental parameters of pervaporation were performed with a downstream pressure of 1500 Pa, with real wastewater: aroma flowrate (*Ji*), permeability (*Li*), and selectivity of each aroma (against water).

Compound	*J_i_* [mol/m^2^.s]	*L_i_* [mol/(m.s.Pa)]	*Separation Factor [–]*
***Sardine cooking wastewater***			
**1-Penten-3-ol**	**6.58 × 10^−7^ ± 8.64 × 10^−9^**	**6.25 × 10^−11^ ± 4.15 × 10^−12^**	**4.20 ± 0.28**
1-Octen-3-ol	3.91 × 10^−8^ ± 1.66 × 10^−9^	2.33 × 10^−11^ ± 7.95 × 10^−13^	11.19 ± 0.38
2-Nonanone	1.89 × 10^−8^ ± 9.67 × 10^−10^	1.48 × 10^−11^ ± 9.17 × 10^−13^	51.03 ± 3.14
Heptanal	2.80 × 10^−8^ ± 9.56 × 10^−10^	3.73 × 10^−12^ ± 3.06 × 10^−13^	7.31 ± 0.60
(*E*2, *Z*6)-Nonadienal	4.65 × 10^−7^ ± 2.86 × 10^−8^	1.48 × 10^−11^ ± 9.17 × 10^−13^	171.15 ± 19.73

The data presented are the mean ± s.d. values. Underlined compounds are off-flavours.

## Data Availability

Not applicable.
